# Soil Bacteriome Shifts along a Cultivation Gradient in Southwestern Spanish Wetlands

**DOI:** 10.1007/s00248-025-02660-8

**Published:** 2025-11-29

**Authors:** José Luis González-Pimentel, Alba Cuecas, Consolación Álvarez, Vicente Mariscal

**Affiliations:** Instituto de Bioquímica Vegetal y Fotosíntesis, Consejo Superior de Investigaciones Científicas and Universidad de Sevilla, Américo Vespucio 49, 41092 Seville, Spain

**Keywords:** Soil bacteriome, Rice paddies, Wetland soils, 16S rRNA gene sequencing, Denitrification

## Abstract

Understanding how long-term agricultural practices affect soil bacteriome is essential for sustainable land management. In the Guadalquivir Marshes of southwestern Spain, which encompass both Doñana National Park and one of Europe’s most productive rice cultivation areas, decades of rice farming have transformed natural wetlands into artificial agroecosystems. Although bacterial degradation in cultivated soils has been previously suggested, comparative analyses between rice paddies and adjacent natural wetlands remain scarce.

Here, we characterized the soil bacteriome across a cultivation gradient by comparing undisturbed natural marshes, within Doñana National Park, with rice fields cultivated for 25 years (Cantarita) and 80 years (Mínima 2). Using full 16S rRNA gene via long-read metabarcoding and standardized soil physicochemical assays, we analysed taxonomic composition, environmental associations, and predicted functional profiles.

Our results reveal a progressive restructuring of bacterial communities with increased cultivation time, notably a significant enrichment of *Chloroflexota* (especially *Anaerolineae*) and a decline in *Actinomycetota* and *Planctomycetota* in paddy soils. Functional predictions indicated a higher potential for denitrification in cultivated soils—likely involving *Chloroflexota* taxa—compared to more diverse nitrogen pathways in natural sites. These shifts were strongly associated with changes in pH, electrical conductivity, calcium carbonate, and nitrate levels. Remarkably, most bacterial differences were already evident within the first 25 years of cultivation, underscoring the rapid ecological impact of intensive rice cultivation.

Notably, we identified specific bacterial groups (*Anaerolineae* and *Nocardioides* in paddy soils; *Euzebya*, *Rubrobacter*, and *Planctomycetota* in natural wetlands), whose enrichment was associated with soil type. This approach highlights the value of integrating bacterial-based assessments into sustainable wetland management strategies.

**Supplementary Information:**

The online version contains supplementary material available at 10.1007/s00248-025-02660-8.

## Introduction

The Lower Guadalquivir wetlands, located in southwestern Spain, represent a unique environmental and socio-economic region in Europe. This area includes Doñana National Park, an ecosystem of exceptional ecological value, and one of the most important agricultural zones in the Iberian Peninsula. Since the 1940s, agricultural expansion—primarily rice farming—has transformed parts of the Guadalquivir Marshes into anthropogenic wetlands. Today, approximately 40,000 hectares are dedicated to rice cultivation in this region, accounting for nearly 40% of Spain’s national rice production and around 10% of the total rice output within the European Union [18, 10, 15]. These farmlands are predominantly managed by small-scale farmers, many of whom have adopted sustainable agricultural practices supported by regional policies and agri-environmental programmes. However, the prolonged and intensive use of these wetlands for rice production may have driven significant ecological transformations, including alterations in the composition and functionality of native soil bacterial communities. Soil microorganisms play a fundamental role in ecosystem functioning and biogeochemical cycling [13, 41]. Intensive agricultural practices and the long-term use of inorganic fertilisers have been shown to disrupt soil microbial communities, leading to reductions in both species richness and diversity [22, 50]. Moreover, continued use of agrochemicals can impair beneficial plant–microbe interactions [25, 44, 45]. Previous studies in the rice paddies of the Lower Guadalquivir region have explored the negative impacts on bulk soil and rhizosphere bacteriomes, as well as the potential of native N₂-fixing cyanobacteria as a sustainable nitrogen source for rice cultivation [23, 24]. However, knowledge remains limited regarding the diversity and structure of soil bacterial communities in these anthropogenic wetlands, particularly in comparison with their natural, non-anthropised counterparts.

Marshland soils across various latitudes in China—currently the most extensively studied region in this context—are predominantly composed of members of the phyla *Pseudomonadota*, *Chloroflexota*, *Actinomycetota*, *Bacteroidota*, and *Acidobacteriota*, regardless of whether the soils originate from natural wetlands or are used as paddy fields [3, 30, 52]. In the Guadalquivir Marshes, a similar phylum-level distribution has been reported, although exclusively in cultivated soils, as the bacterial composition of natural marshlands in this area has not yet been characterised [24]. When comparing paddy and natural soils, results vary among studies,however, a commonly observed trend is the enrichment of bacteria belonging to the class *Anaerolineae* (phylum *Chloroflexota*) in paddy soils. This taxon is associated with anaerobic carbon metabolism and reflects the anoxic conditions prevalent in flooded rice paddies [27, 28, 31].

Another key question is how soil bacteriome evolves over extended periods of intensive crop cultivation. Current research has mainly focused on comparative analyses of bacterial communities and activity across plots with differing cultivation histories [2]. However, such studies remain limited and are largely concentrated in specific regions of China. The temporal scales examined range from a few decades [52] to several millennia [30]. Liu et al. [30] reported a gradual decline in bacterial taxa affiliated with the phyla *Pseudomonadota*, *Bacteroidota*, and *Actinomycetota*, alongside a concurrent increase in *Acidobacteriota* and *Verrucomicrobiota* in paddy soils. Similarly, Zhang et al. [52] observed incremental shifts in the relative abundances of *Acidobacteriota* and *Chloroflexota* across rice paddies subjected to different durations of cultivation. These findings suggest that prolonged agricultural use may drive progressive changes in bacterial community composition, potentially impacting soil health and ecosystem functionality. Nonetheless, while certain bacterial trends may emerge consistently across sites, others are likely shaped by local environmental conditions and cultivation histories, underscoring the need for site-specific assessments.

Scientific consensus increasingly recognises the critical role of the soil microbiome in assessing soil health, particularly in agricultural systems [12]. A key objective in this field is the identification of bacterial bioindicators that signal soil dysbiosis. For example, a higher prevalence of spore-forming and stress-tolerant Gram-positive bacteria such as *Actinomycetota* has been associated with environmental stress [5]. However, it is important to acknowledge that there is no universally ‘optimal’ soil or definitive set of ideal attributes,rather, soil health is inherently context-dependent and varies across ecosystems [16]. Several studies have demonstrated strong correlations between bacterial community composition and land-use types, showing that bacteriome data can be used to predict both physicochemical variables and overall soil quality [21]. In this regard, inferring soil health status from bacteriome profiles requires the establishment of robust baselines through the characterisation of bacterial communities in relatively undisturbed, natural soils and their comparison with those in degraded or anthropogenically impacted environments [40, 49]. From a technical perspective, the field continues to advance, with next-generation sequencing (NGS) technologies offering significant potential for improving the resolution and applicability of microbiome-based soil health assessments [16].

The Guadalquivir wetlands offer a unique setting for investigating the effects of different levels of anthropogenic impact on soil prokaryotic bacteriome, as they encompass both the relatively undisturbed soils of Doñana National Park and one of the Europe’s largest rice cultivation areas, characterised by continuous irrigation and intensive nitrogen fertilisation [26]. In this region, balancing the conservation of natural wetland ecosystems and their biodiversity with the demands of agricultural productivity is of particular importance. Accordingly, monitoring human-induced changes is essential for achieving sustainable land management. Addressing these challenges requires not only comparative analyses between natural and cultivated sites, but also an understanding of how the duration and intensity of agricultural practices shape soil bacterial dynamics across a temporal gradient.

In this study, we evaluate the impact of anthropogenic activity on the soils of the Guadalquivir Marshes by comparing the prokaryotic bacteriome of natural wetlands with those of rice paddies established at different historical stages. Specifically, we contrast undisturbed soils from Doñana National Park with long-term agricultural sites located at Mínima 2 (80 years of cultivation) and more recently cultivated Cantarita (25 years), to assess the agricultural impact on soil bacterial communities. Through a combined molecular and physicochemical approach, including 16S rRNA gene sequencing via the PacBio CCS amplicon data and standardised soil assays, we aim to: (i) identify taxonomic shifts associated with cultivation; (ii) elucidate the interplay between bacteriome composition and soil properties in reshaping wetland ecosystems under intensive rice cultivation; and (iii) propose potential bacterial indicators for future soil health monitoring. Our findings contribute essential baseline data for assessing agricultural impacts and ecological health in analogous wetland systems using bacteriome-based approaches.

## Methods

### Sample Collection and Pre-processing

Soil sampling was conducted in the seasonal Guadalquivir Marshes, located in southwestern Spain. Three sites representing distinct cultivation histories were selected: (i) undisturbed soils from Doñana National Park, (ii) Mínima 2, a rice-cultivated area with approximately 80 years of continuous use, and (iii) Cantarita, a rice field cultivated for around 25 years (Supplementary Table S1 and Supplementary Fig. 1). Sampling took place in mid-April 2024, prior to the onset of the rice-growing season, thus, undisturbed soil samples from the three sites were analysed.

Five samples were collected from each study site, with their locations detailed in Supplementary Table S1 and Supplementary Fig. 1. Each sample consisted of a composite soil sample obtained by pooling material from five individual 5 × 5 cm surface footprints spaced approximately 50 cm to 1 m apart. These subsamples were homogenized to ensure representativeness. Soil was collected from the top 5–10 cm of the profile, targeting the biologically active layer. All samples were immediately stored at 4 °C during transport to the laboratory. Upon arrival, sub-samples were subdivided for subsequent analyses. A 50 g aliquot of each sample was flash-frozen in liquid nitrogen and stored at − 70 °C for DNA extraction.

### DNA Extraction and Sequencing

Small aliquots (250–300 mg) from each sample were used for DNA extraction using the DNeasy PowerSoil Pro Kit (Qiagen, Germany; Cat. No. 47014), following the manufacturer’s protocol. DNA concentrations were quantified by fluorometry using the Qubit™ dsDNA HS Assay Kit (Invitrogen, USA). Amplification and sequencing of the 16S rRNA gene were performed by the Foundation for the Promotion of Health and Biomedical Research of the Valencia Region (FISABIO, Valencia, Spain) using the Sequel II PacBio system (Pacific Biosciences, Menlo Park, CA, USA). Full-length 16S rRNA genes were amplified from the extracted genomic DNA using primers 27F (AGRGTTYGATYMTGGCTCAG) and 1492R (RGYTACCTTGTTACGACTT). PCR amplification was carried using the KAPA HiFi Hot Start DNA Polymerase (KAPA Biosystems), according to [8]. The library was prepared according to the manufacturer’s protocol described in Part Number 101–599–700, Version 04 (PacBio). The Sequel II PacBio system was used for sequencing, employing the Sequel II Sequencing Kit 2.0 (PacBio, USA).

### Physicochemical Analysis of Soil Parameters

Approximately 800 g of soil was air-dried and sieved through a 2 mm mesh prior to physicochemical analysis. Soil pH was measured in a 1:2.5 (w/v) suspension of soil in 1 M KCl after 30 min of shaking. Total nitrogen (N) was determined by dry combustion in an elemental analyzer Primacs SNC 100 IC-E Skalar by combustion of the sample [47]. Available phosphorus was extracted with 0.5 M NaHCO_3_ (pH 8.5) according to Olsen & Sommers [35] and measured colorimetrically by an AutoAnalyzer Bran Luebbe AA III. Soils were extracted with KCl 1 M for determining N-NH_4_^+^ and N-NO_3_^−^ by an AutoAnalyzer Bran Luebbe AA III. The electrical conductivity (EC) of the soil was measured in a 1:5 sample/H_2_O, after shaking for 1 h, with a CRISON MultiMeter MM 41.

### Bioinformatic Analysis

Raw sequence data were processed using QIIME2 version 2024.2 [6]. Demultiplexing, quality filtering, chimera detection, and clustering were performed using the DADA2 and denoise-ccs plugins [9]. Amplicon sequencing variants (ASVs) were taxonomically classified using the q2-feature-classifier plugin [7], employing the Naïves-Bayes classifier against the SILVA 16S rRNA reference database (version 138.1,[39]. ASVs with ≥ 10 sequences or observed in ≥ 2 samples were retained.

Feature tables were analysed using the microeco R package (v1.12.0; [32]), which utilises an R6 class data structure. Differential abundance analysis at the family level was conducted using an Analysis of compositions of microbiomes with bias correction (ANCOM-BC2) on the non-rarefied ASV count table (Lin and Peddada 2020 [29]), with studied areas as a fixed factor. For each group, the global (omnibus) Wald test across sites and pairwise contrasts with bias-corrected log-fold changes (LFCs) were applied. P-values were adjusted using Benjamini–Hochberg FDR. The ‘rf’ method based on random forest and non-parametric testing [4, 51] was calculated as an exploratory multivariate check of separability. After rarefaction of samples with lowest sequences, Alpha diversity metrics, including Observed ASVs, Shannon and Simpson indices and Pielou’s evenness [37, 42, 43] were calculated. Statistical comparison between sampling areas with both parametric analysis of variance (ANOVA), and non-parametric Wilcoxon rank sum test using Benjamini-Hochberg (BH) adjustment for false discovery rate (FDR) corrections.

Canonical correspondence analysis (CCA) at the genus level, integrating physicochemical parameters, was performed using the vegan R package v.2.6–8 [34]. In addition, a permutation test was developed to evaluate the significance of the correlation between the communities at the genus level and the physicochemical parameters. Co-occurrence network analysis of ASVs was conducted based on Spearman’s correlation (r > 0.8) using igraph v.2.0.3 across the three study areas. All statistical tests were validated at α = 0.05.

Functional profile prediction was performed using PICRUSt2 (v2.6.0), with the updated PICRUSt2-SC reference database based on the Genome Taxonomy Database (GTDB r214; [14, 36]. Differences in KO term abundances were assessed using the non-parametric Kruskal–Wallis test, with p-values adjusted for multiple comparisons using the false discovery rate (FDR) method. Post hoc pairwise comparisons were performed using Dunn’s test, with p-values corrected using the Benjamini–Hochberg (BH) method. Raw data have been deposited in the NCBI Sequence Read Archive (SRA) under accession numbers SRX28922929–SRX28922943.

## Results

**Fig. 1:**
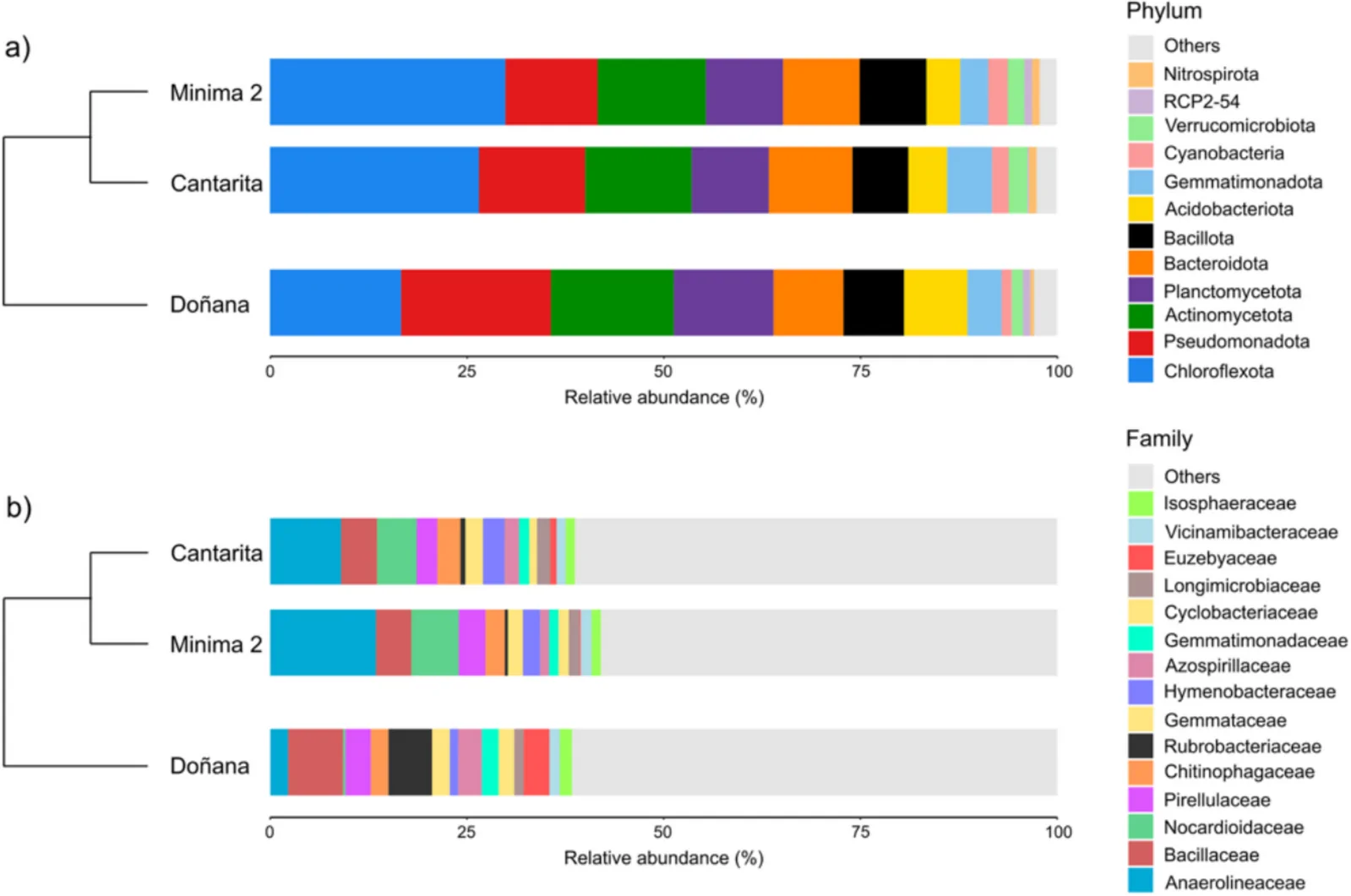
Relative abundance of major bacterial phyla and families across the three study sites: Doñana National Park (natural wetland), Cantarita (25 years of rice cultivation), and Mínima 2 (80 years of rice cultivation). Data are based on 16S rRNA gene sequencing. “Others” denote taxa below 1% abundance in at least one study site

A total of 699,725 raw DNA sequences (mean length: 1490 bp) were obtained from fifteen soil samples. After quality filtering and trimming, denoising yielded a total of amplicon sequence variants (ASVs) ranging from 429 to 5,485 per sample (Table 1). Taxonomic assignment revealed that soil bacterial communities across the three study sites were dominated by members of the phyla *Chloroflexota* (16.65–29.92%), *Pseudomonadota* (17.29–18.45%), and *Actinomycetota* (11.72–19.04%) (Fig. 1a). Other phyla with lower relative abundances included *Planctomycetota* (9.80–12.69%), *Bacteroidota* (8.88–10.64%), *Bacillota* (7.09–8.47%), *Acidobacteriota* (4.29–8.05%), and *Gemmatimonadota* (3.59–5.70%). The archaea content in samples (< 0.1%), was considered negligible and, therefore, omited in the subsequent analyses.

**Table 1:** Summary of raw and filtered sequences, and number of observed ASVs per sample

No. of sample^1^	No. of raw sequences	No. of filtered sequences	No. of observed ASVs
M-1	51483	19313	1203
M-2	24528	6252	449
M-3	27594	7851	596
M-4	27978	9525	776
M-5	30086	8845	741
CA-1	27092	7680	599
CA-2	23306	9503	571
CA-3	23571	7915	555
CA-4	53530	22972	1425
CA-5	27960	8973	655
PN-1	22968	7285	567
PN-2	21452	5682	429
PN-3	23423	8573	563
PN-4	23376	8501	717
PN-5	291378	142021	5485
Total	699725	280891	15331

^1^ M: Mínima 2 (80 years of cultivation history); CA: Cantarita (25 years of cultivation history); PN: (Doñana National Park)

A clear gradient in community structure was observed from the natural wetland soils in Doñana to the oldest cultivated site, Mínima 2, with Cantarita representing an intermediate (less ancient) stage of cultivation. The relative abundance of *Chloroflexota* increased markedly along this gradient (16.65 ± 8.27% in Doñana, 26.58 ± 10.31% in Cantarita, and 29.92 ± 5.08% in Mínima 2), whereas *Actinomycetota* declined progressively (19.04 ± 4.99% in Doñana, 13.48 ± 8.17% in Cantarita, and 11.72 ± 2.86% in Mínima 2). Moderate reductions were also observed for *Planctomycetota* (12.69 ± 3.45% in Doñana; 9.82 ± 3.43% in Cantarita; 9.80 ± 1.64% in Mínima 2) and *Acidobacteriota* (8.05 ± 2.95% in Doñana; 4.93 ± 1.12% in Cantarita; 4.29 ± 1.54% in Mínima 2). Although *Acidobacteriota* represented a smaller fraction of the total community, its relative abundance was nearly halved along the cultivation history intensification gradient, indicating a consistent and potentially sensitive response. In contrast, *Bacteroidota* was more representative in cultivated soils (10.64 ± 8,79% in Cantarita and 9.75 ± 2.53% in Mínima 2) compared to the non-cultivated site (8.88 ± 4.82%).

**Fig. 2:**
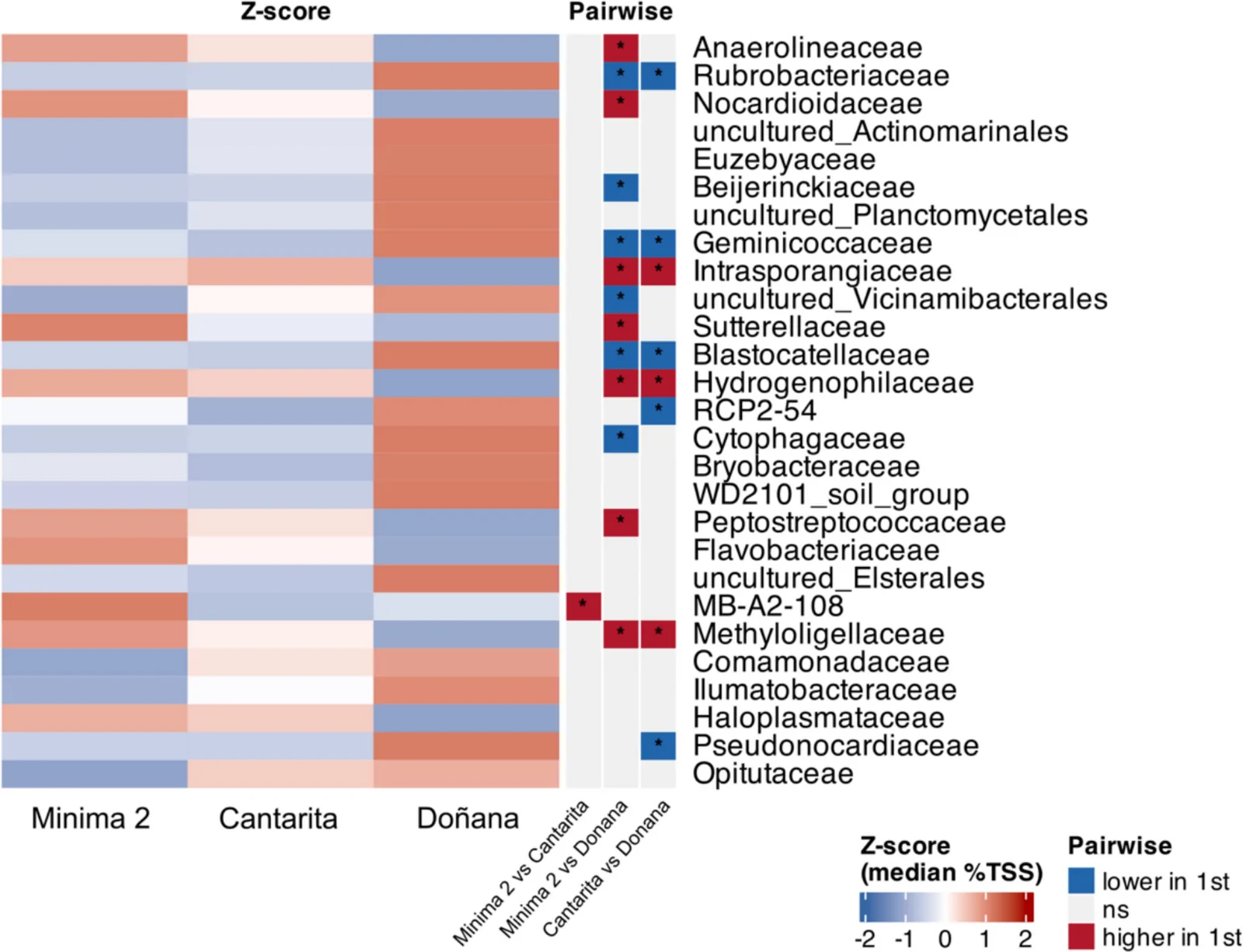
Analysis of compositions of microbiomes with bias correction (ANCOM-BC2 omnibus test, σ < 0.05) with median relative abundance (%TSS) by site at the family level and pairwise differences significant after BH/FDR (σ < 0.05)

At the family level, distinct differences in the relative abundance of dominant taxa were observed across the three study areas, showing a change in abundance of specific groups such as *Anaerolineaceae* and *Nocardioidaceae* in Mínima 2 and Cantarita, and *Rubrobacteriaceae* and *Euzebyaceae* in Doñana (Fig. 1b). Microorganisms affiliated to *Anaerolineaceae* (*Chloroflexota*) and *Nocardioidaceae* (*Actinomycetota*) showed a higher relative abundance in cultivated soils (13.44 ± 1.41% and 6.01 ± 1.81%, for Minima 2; 9.00 ± 4.63% and 5.02 ± 4.58%, for Cantarita, respectively) than in the natural site of Doñana (2.26 ± 1.22% and 0.29 ± 0.25%, respectively). Differential abundance analysis revealed a significant increase of these groups in Mínima 2, the oldest cultivated site, compared to Doñana (Fig. 2 and Supplementary Figures S2). These results were confirmed by ‘rf’ analysis (Supplementary Figure S3). In contrast, *Rubrobacteriaceae* and *Euzebyaceae* (both within *Actinomycetota*) exhibited the opposite trend, being more abundant in the natural soils of Doñana (5.54 ± 2.36% and 3.27 ± 2.80%, respectively) than in Mínima 2 (0.39 ± 0.26% and 0.16 ± 0.22%, respectively) and Cantarita (0.63 ± 0.30% and 0.80 ± 0.22%, respectively).

**Fig. 3:**
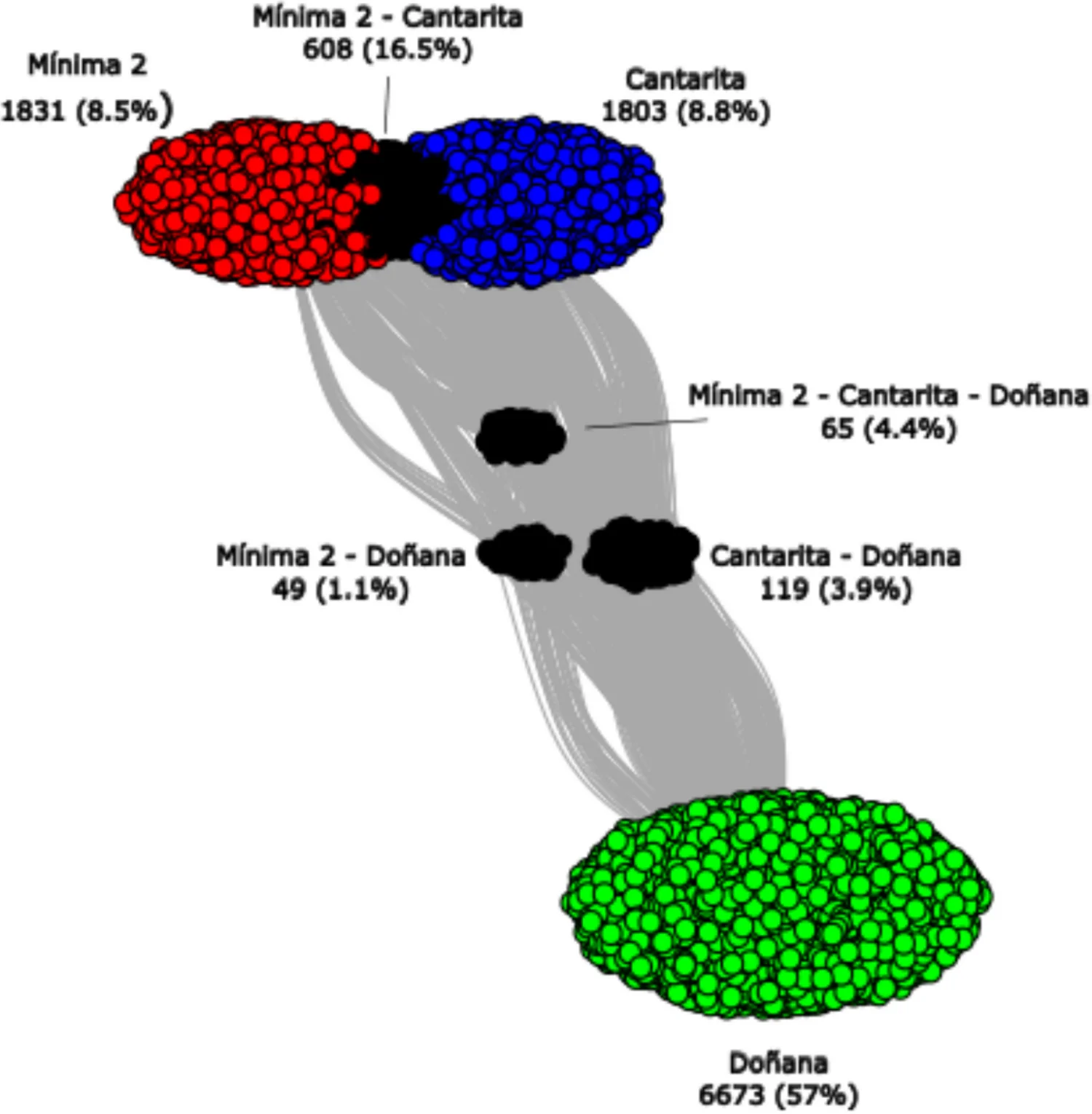
Co-occurrence network of ASVs across the three study sites. Shared and exclusive ASVs highlight community divergence associated with cultivation history. Core taxa shared across all sites are indicated. In green, ASVs from Doñana, in red, ASVs from Minima 2, in blue, ASVs from Cantarita

To visualise patterns of similarity and divergence among bacterial communities across sites, we performed a co-occurrence network analysis at the ASV level (Fig. 3). Mínima 2 and Cantarita clustered closely, sharing 16% of ASVs exclusively, and were clearly separated from Doñana. This pattern supports a directional shift in bacterial community structure associated with agricultural intensification. As expected, Doñana and Cantarita shared more exclusive ASVs (3.9%) than Doñana and Mínima 2 (1.1%), reflecting the longer cultivation history of the latter. Only 4.4% of ASVs were shared across all three sites, representing a core bacteriome likely composed of resilient taxa. These included members of major phyla such as *Actinomycetota* (*Euzebyaceae*, *Mycobacteriaceae*, *Rubrobacteraceae*), *Bacillota* (*Bacillaceae*), *Bacteroidota* (*Chitinophagaceae*, *Hymenobacteraceae*, *Rhodothermaceae*), *Chloroflexota* (*Anaerolineaceae*, uncultured *Ardenticatenales*), *Planctomycetota* (*Pirellulaceae*), and *Pseudomonadota* (*Defluviicoccaceae*). Although differences were observed in bacterial composition between samples, Observed ASVs, Shannon and Simpson Alpha diversity indices did not show any significant variation among samples. In contrast, Pielou’s evenness was significantly lower in Doñana compared to Mínima 2 and Cantarita, indicating greater unevenness in the natural wetland communities (Supplementary Figure S4 and Supplementary Figure S5).

Physicochemical profiling revealed marked significative differences among the study sites in pH, electrical conductivity, CaCO₃ content, total carbon, and nitrate concentration (Supplementary Figure S6, Supplementary Table S2). Soils from Doñana exhibited significantly higher pH values compared to the cultivated soils, and pH was negatively correlated with electrical conductivity, total carbon, carbonate content, and nitrate levels. Among all measured parameters, pH emerged as the most distinctive feature of the natural wetland soils. In contrast, electrical conductivity was elevated in cultivated sites and positively correlated with both nitrate and carbonate concentrations. Carbonate levels also showed a positive correlation with total carbon, which was more abundant in the cultivated sites than in Doñana.

**Fig. 4:**
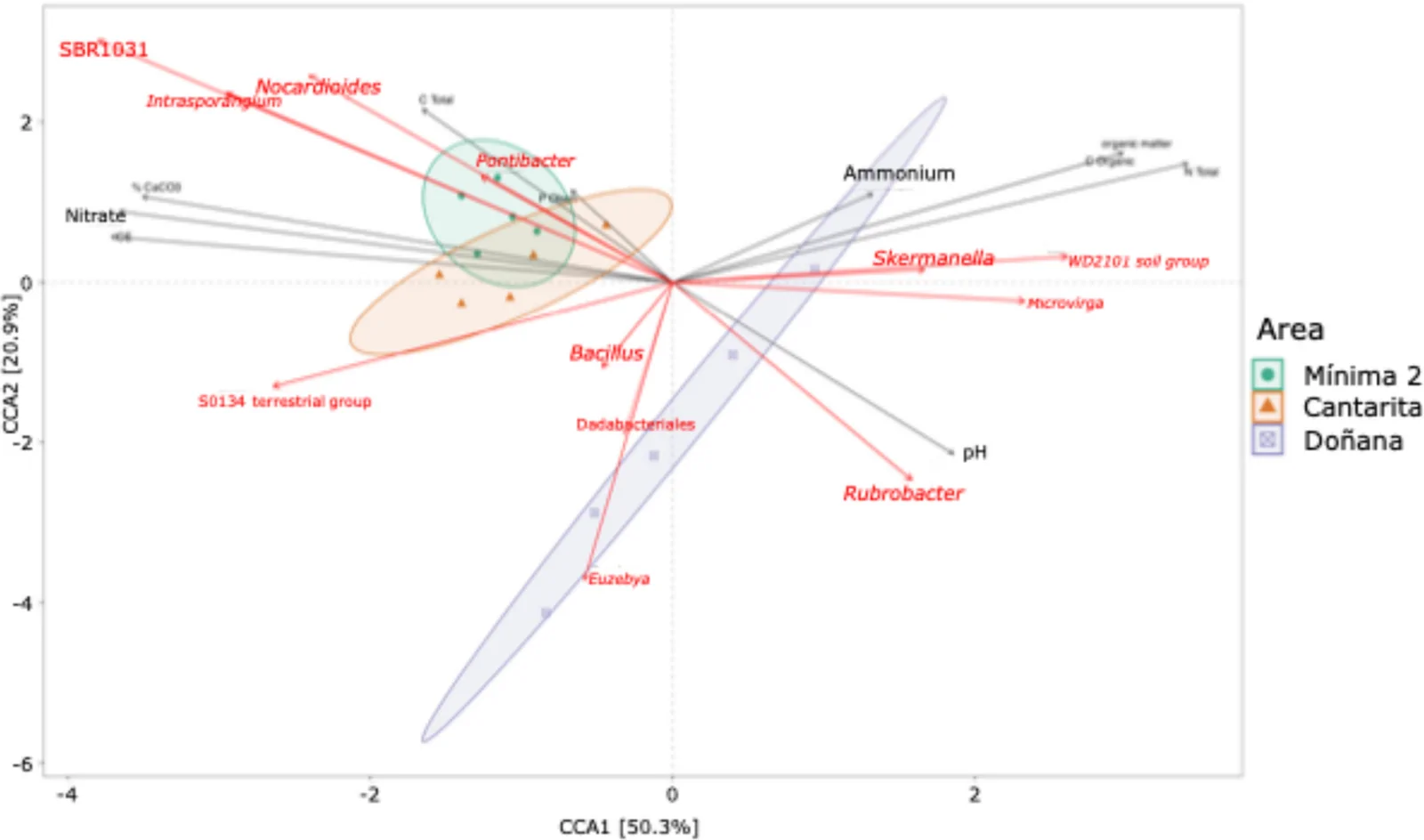
Canonical correspondence analysis (CCA). Genus-level bacterial composition is represented with red arrows and soil physicochemical parameters are represented with black arrows. Samples from cultivated sites cluster separately from those of Doñana, with pH and nitrate levels driving community separation

Canonical correspondence analysis (CCA), integrating genus-level taxonomic data with soil parameters, revealed a clear separation between samples from the natural site (Doñana) and those from the cultivated areas (Mínima 2 and Cantarita), which showed partial overlap (Fig. 4). Greater heterogeneity was observed among Doñana samples, while cultivated sites exhibited tighter clustering. Taxa enriched in Doñana included the WD2101 soil group (*Planctomycetota*), *Euzebya* and *Rubrobacter* (*Actinomycetota*), *Microvirga* and *Skermanella* (*Pseudomonadota*), Dadabacteriales (*Candidatus* Dadabacteria), and *Bacillus* (*Bacillota*). Permutation test showed an inverse correlation among *Candidatus* Dadabacteriales and *Euzebya* with Total C, Organic C, Organic matter and Total N (Supplementary Figure S7). In cultivated soils, any of the groups showed significative changes. However, taxa most strongly associated with cultivated soils included SBR1031, *Nocardioides*, and *Intrasporangium* (all *Actinomycetota*), the latter not previously highlighted in other analyses. Among environmental variables, electrical conductivity, nitrate concentration, CaCO₃ content, and, to a lesser extent, total carbon contributed most to the separation of cultivated sites.

**Fig. 5:**
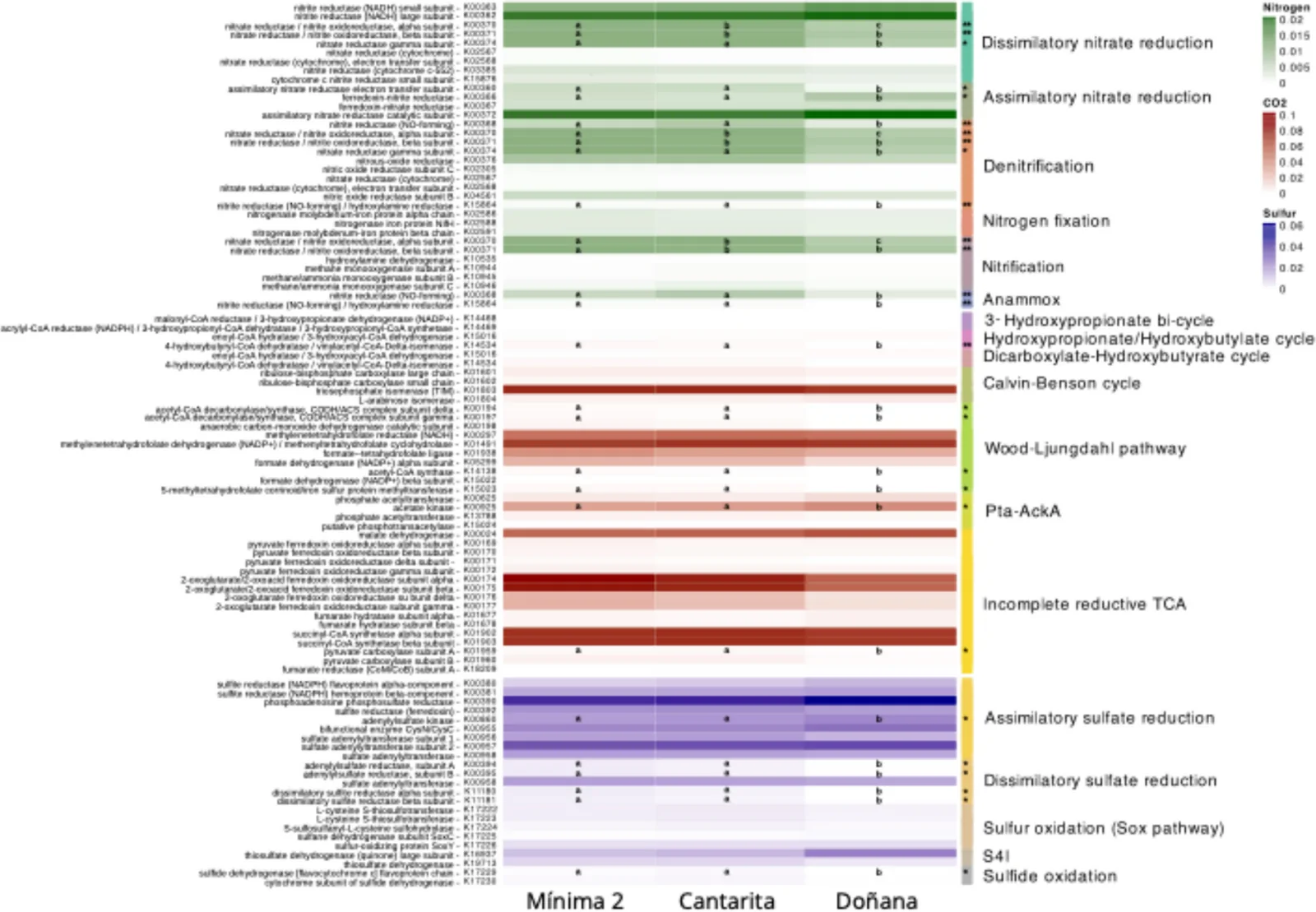
Predicted functional profiles of nitrogen metabolism pathways across the three study sites. In green, pathways involved in the nitrogen biogeochemical cycle; in red, pathways involved in the carbon cycle; in purple, pathways involved in the sulphur biogeochemical cycle. Values indicate relative abundance with respect to the total classified ASVs. KEGG terms of each pathway are indicated. Asterisks indicate significance of KO terms calculated with Kruskal–Wallis test; ρ = 0.01 (**), ρ = 0.05 (*). “a”, “b”, “c” letters denote distinction among studied areas after Dunn’s test for pairwise comparison

Predicted metabolic profiles revealed a clear functional differentiation between the cultivated areas (Mínima 2 and Cantarita) and the natural wetland of Doñana (Fig. 5). Nitrogen metabolic pathways including dissimilatory nitrate reduction, denitrification, nitrification and anammox differed significantly between cultivated and undisturbed as determined by Kruskal–Wallis and post hoc pairwise Dunn tests (Fig. 5). The alpha and beta subunits of nitrate reductase/nitrite oxidoreductase (K00370, Z = –4.14; K00371, Z = –4.01; both Mínima 2 > Doñana, ρ < 0.001), which participate in dissimilatory nitrate reduction, denitrification, and nitrification, were significantly more abundant in cultivated soils. For dissimilatory nitrate reduction, the gamma subunit of nitrate reductase (K00374; Z = –3.66, Mínima 2 > Doñana, ρ < 0.001) also showed increased abundance. In the denitrification and anammox pathways, nitrite reductase (NO-forming) (K00368; Z = –3.96, Cantarita > Doñana, ρ < 0.001) and nitrite reductase NO-forming/hydroxylamine reductase (K15864; Z = –4.31, Mínima 2 > Doñana, ρ < 0.001) were differentially represented across the three sites. In contrast, Doñana soils exhibited higher predicted levels of ferredoxin-nitrite reductase (K00366; Z = –3.18, Doñana > Cantarita, ρ = 0.002), associated with assimilatory nitrate reduction. Although less pronounced, significative differences were also observed in KO terms for carbon and sulphur metabolism. In particular, enzymes linked to dissimilatory sulphate reduction were more abundant in cultivated soils, suggesting broader functional shifts potentially driven prolonged anaerobic conditions characteristic of rice paddy soils.

## Discussion

Our findings demonstrate that long-term rice cultivation in the wetland soils of the Guadalquivir Marshes induces pronounced shifts in soil bacterial communities. Taxonomic and functional analyses consistently differentiated cultivated sites (Mínima 2 and Cantarita) from the original, natural wetland (Doñana), with community changes correlating with cultivation duration. These shifts were evident across multiple taxonomic levels, including phylum and family composition, indicator taxa, co-occurrence network structure, and predicted nitrogen metabolism pathways, all closely linked to specific environmental parameters. Collectively, these results underscore the influence of soil cultivation on bacterial community structure and function. To our knowledge, this is the first study to simultaneously characterise both natural and intensively managed wetland bacteriomes in southwestern Europe, providing a valuable baseline for future ecological monitoring and sustainable land management. Previous work in the Guadalquivir Marshes identified *Chloroflexota* as the dominant phylum in paddy soils [24]. Consistent with these findings, our study revealed a progressive enrichment of *Chloroflexota* taxa with increasing cultivation duration, particularly members of the class *Anaerolineae*, including the family *Anaerolineaceae* and the taxon SBR1031. Although *Chloroflexota* is not typically dominant in comparable studies from China, enrichment of specific lineages within this phylum has been reported [27, 28, 30], with some studies linking their abundance to cultivation history [52]. Environmental correlations further support this trend: members of *Anaerolineae* have been negatively correlated with soil pH [30], while *Chloroflexota* abundance has shown positive correlations with electrical conductivity, organic matter, and total nitrogen [52]. Our results align with these patterns, with additional positive correlations observed for nitrate and calcium carbonate. Members of *Anaerolineae* are known for their metabolic versatility, including the degradation of a wide range of carbon substrates and involvement in nitrogen cycling, particularly nitrite reduction [17]. These traits suggest that *Chloroflexota*—especially *Anaerolineae*—may serve not only as indicators of paddy soils, but also as key contributors to carbon and nitrogen biogeochemical processes in these ecosystems. It is worth noting the high values of available Phosphorous found in the cultivated soils (Supplementary Table 3). Land conversion for agricultural use involved the use of large amounts of phosphogypsum to amend the high salinity of the soils around the 1970s [1], which led to the significant differences observed in the physicochemical parameter analyses.

The phylum *Actinomycetota*, identified as a core component of soil bacterial communities in both our study and that of Iniesta et al. [24], exhibited a marked decline in paddy soils. Although this phylum has also been detected in Chinese paddy fields, it is generally less dominant [29, 30, 52]. Interestingly, Liu et al. [30] reported an increase in *Actinomycetota* abundance with prolonged cultivation; however, their study encompassed chronosequences spanning centuries to millennia of rice cultivation, suggesting that such shifts may emerge only after extended agricultural use. Despite the overall decline observed in our sites, the genus *Nocardioides* remained relatively abundant in cultivated soils. This genus is known for its capacity to degrade a wide range of organic pollutants and has been isolated from paddy environments, suggesting a potential role in bioremediation [33]. In contrast, natural soils from Doñana were enriched in *Rubrobacter* and *Euzebya*, two *Actinomycetota* genera frequently co-occurring in extreme environments [19]. *Rubrobacter* is recognised for its resistance to high temperatures and low water or nutrient availability, while *Euzebya* may be favoured by the slightly higher pH and more variable salinity and temperature conditions observed in Doñana. In mid-latitude regions such as the Guadalquivir Marshes, summer droughts can elevate surface soil temperatures above 50 °C, potentially promoting the proliferation of thermotolerant or moderately thermophilic taxa [20]. Another key taxonomic distinction was the higher relative abundance of *Planctomycetota* in the natural marshes of Doñana, a pattern also reported in non-cultivated mudflats in China [52]. The reduced representation of this phylum in paddy soils may reflect its sensitivity to altered redox conditions and nutrient availability. In fact, rice paddies are kept intentionally flooded for long, continuous periods, sustaining low redox conditions in the bulk soil. By contrast, marshes undergo natural wet–dry and tidal cycles that periodically re-oxygenate sediments and create spatially patchy oxic microsites. The stable waterlogging in paddies favours anaerobic metabolisms, whereas marsh dynamics limit the persistence of anoxia. Most *Planctomycetota* taxa are involved in nitrogen cycling under oxic or microoxic conditions, which are likely diminished in water-saturated, fertilised soils [48]. Under anoxic conditions, some members can fix nitrogen using ammonium or nitric oxide (NO) as substrates [11], but their ecological roles may be constrained in intensively managed systems. These observations align with our functional profile predictions, which indicated enhanced denitrification potential in cultivated soils—likely mediated by *Chloroflexota*—and increased production of nitrous oxide (N₂O), a potent greenhouse gas commonly emitted from rice paddies [38]. In contrast, natural soils favoured alternative nitrogen pathways, including assimilatory and dissimilatory nitrate reduction, potentially limiting N₂O emissions. These findings underscore the importance of agricultural strategies that not only sustain crop productivity but also promote bacterial communities capable of mitigating greenhouse gas emissions and supporting long-term soil health.

In conclusion, although this study only encompasses three different soils with a limited number of samples, our study provides evidence that long-term rice cultivation in the Guadalquivir Marshes has led to compositional and functional restructuring of soil bacterial communities. Taxonomic patterns across multiple levels indicate a shift towards groups adapted to anaerobic conditions, high nutrient availability, and altered nitrogen cycling. By comparing sites with different cultivation histories and integrating taxonomic and functional analyses, we identified bacterial taxa that were enriched in cultivated soils. Notably, most community shifts were already apparent within the first 25 years of cultivation, highlighting the rapid ecological response of soil bacteriomes to agricultural intensification. As emphasised by Timmis and Ramos [46], realising the full potential of bacteriome-based diagnostics will require long-term investment in coordinated monitoring frameworks, supported by advanced molecular tools and comprehensive reference datasets.

## Supplementary Information

Below is the link to the electronic supplementary material.

Supplementary file1 (DOCX 5979 KB)

## Data Availability

Raw data have been deposited in the NCBI Sequence Read Archive (SRA) under accession numbers SRX28922929–SRX28922943.

## References

[1] Abril JM, García-Tenorio R, Enamorado SM, Hurtado MD, Andreu L, Delgado A (2008) The cumulative effect of three decades of phosphogypsum amendments in reclaimed marsh soils from SW Spain: (226)Ra, (238)U and Cd contents in soils and tomato fruit. Sci Total Environ 15(1–3):80–88. 10.1016/j.scitotenv.2008.05.013 10.1016/j.scitotenv.2008.05.013 18602676

[2] Addesso R, Araniti F, Bloise A et al (2025) Soil organic matter quality in an olive orchard differently managed for 21 years: insights into its distribution through soil aggregates and depth. Agric Ecosyst Environ 380:109388. 10.1016/j.agee.2024.109388

[3] Ahn JH, Lee SA, Kim JM, Kim MS, Song J, Weon HY (2016) Dynamics of bacterial communities in rice field soils as affected by different long-term fertilisation practices. J Microbiol 54:724–731. 10.1007/s12275-016-6463-3 27796926 10.1007/s12275-016-6463-3

[4] Beck D, Foster JA (2014) Machine learning techniques accurately classify microbial communities by bacterial vaginosis characteristics. PLoS One 9:e87830. 10.1371/journal.pone.0087830 24498380 10.1371/journal.pone.0087830 PMC3912131

[5] Bhaduri D, Sihi D, Bhowmik A, Verma BC, Munda S, Dari B (2022) A review on effective soil health bioindicators for ecosystem restoration and sustainability. Front Microbiol 13:938481. 10.3389/fmicb.2022.938481 36060788 10.3389/fmicb.2022.938481 PMC9428492

[6] Bolyen E, Rideout JR, Dillon MR et al (2019) Reproducible, interactive, scalable and extensible microbiome data science using QIIME 2. Nat Biotechnol 37:852–857. 10.1038/s41587-019-0209-9 31341288 10.1038/s41587-019-0209-9 PMC7015180

[7] Bokulich NA, Kaehler BD, Rideout JR et al (2018) Optimising taxonomic classification of marker-gene amplicon sequences with QIIME 2’s q2-feature-classifier plugin. Microbiome 6:90. 10.1186/s40168-018-0470-z 29773078 10.1186/s40168-018-0470-z PMC5956843

[8] Buetas E, Jordán-López M, López-Roldán A et al (2024) Full-length 16S rRNA gene sequencing by PacBio improves taxonomic resolution in human microbiome samples. BMC Genomics 25:310. 10.1186/s12864-024-10213-5 38528457 10.1186/s12864-024-10213-5 PMC10964587

[9] Callahan BJ, McMurdie PJ, Rosen MJ et al (2016) DADA2: High-resolution sample inference from Illumina amplicon data. Nat Methods 13:581–583. 10.1038/nmeth.3869 27214047 10.1038/nmeth.3869 PMC4927377

[10] Castillo-Manzano JI, Castro-Nuño M, López-Valpuesta L, del Pozo-Barajas R (2021) *Estudio del impacto económico y social del cultivo del arroz en las Marismas del Guadalquivir como dinamizador de la economía andaluza*. Junta de Andalucía, Sevilla

[11] Cuecas A, Barrau MJ, González JM (2024) Microbial divergence and evolution: the case of anammox bacteria. Front Microbiol 15:1355780. 10.3389/fmicb.2024.1355780 38419632 10.3389/fmicb.2024.1355780 PMC10900513

[12] Dahiya UR, Das J, Bano S (2022) Biological indicators of soil health and biomonitoring. In: Malik JA (ed) *Advances in Bioremediation and Phytoremediation for Sustainable Soil Management*. Springer, Cham. 10.1007/978-3-030-89984-4_21

[13] Domeignoz-Horta LA, Pold G, Liu XJA, Frey SD, Melillo JM, DeAngelis KM (2020) Microbial diversity drives carbon use efficiency in a model soil. Nat Commun 11:3684. 10.1038/s41467-020-17502-z 32703952 10.1038/s41467-020-17502-z PMC7378083

[14] Douglas GM, Maffei VJ, Zaneveld JR et al (2020) PICRUSt2 for prediction of metagenome functions. Nat Biotechnol 38:685–688. 10.1038/s41587-020-0548-6 32483366 10.1038/s41587-020-0548-6 PMC7365738

[15] European Commission (2020) A farmer’s toolbox for integrated pest management. https://agriculture.ec.europa.eu/document/download/200c1c37-a76e-4901-b847-276249f23bf2_en?filename=case-study-ipm-spain_en.pdf

[16] Fierer N, Wood SA, de Mesquita CPB (2021) How microbes can, and cannot, be used to assess soil health. Soil Biol Biochem 153:108111. 10.1016/j.soilbio.2020.108111

[17] Freches A, Fradinho JC (2024) The biotechnological potential of the Chloroflexota phylum. Appl Environ Microbiol 90:e01756-23. 10.1128/aem.01756-23 38709098 10.1128/aem.01756-23 PMC11218635

[18] Gómez de Barreda D, Pardo G, Osca JM et al (2021) An overview of rice cultivation in Spain and the management of herbicide-resistant weeds. Agronomy 11:1095. 10.3390/agronomy11061095

[19] González-Pimentel JL, Martín-Pozas T, Jurado V et al (2023) The marine bacterial genus *Euzebya* is distributed worldwide in terrestrial environments: a review. Appl Sci 13:9644. 10.3390/app13179644

[20] González JM, Portillo MC, Piñeiro-Vidal M (2015) Latitude-dependent underestimation of microbial extracellular enzyme activity in soils. Int J Environ Sci Technol 12:2427–2434. 10.1007/s13762-014-0635-7

[21] Hermans SM, Buckley HL, Case BS et al (2020) Using soil bacterial communities to predict physicochemical variables and soil quality. Microbiome 8:79. 10.1186/s40168-020-00858-1 32487269 10.1186/s40168-020-00858-1 PMC7268603

[22] Huang R, McGrath SP, Hirsch PR et al (2019) Plant–microbe networks in soil are weakened by century-long use of inorganic fertilisers. Microb Biotechnol 12:1464–1475. 10.1111/1751-7915.13487 31536680 10.1111/1751-7915.13487 PMC6801139

[23] Iniesta-Pallarés M, Álvarez C, Gordillo-Cantón FM et al (2021) Sustaining rice production through biofertilisation with N₂-fixing cyanobacteria. Appl Sci 11:4628. 10.3390/app11104628

[24] Iniesta-Pallarés M, Brenes-Álvarez M, Lasa AV et al (2023) Changes in rice rhizosphere and bulk soil bacterial communities in the Doñana wetlands at different growth stages. Appl Soil Ecol 190:105013. 10.1016/j.apsoil.2023.105013

[25] Jacoby R, Peukert M, Succurro A et al (2017) The role of soil microorganisms in plant mineral nutrition—current knowledge and future directions. Front Plant Sci 8:1617. 10.3389/fpls.2017.01617 28974956 10.3389/fpls.2017.01617 PMC5610682

[26] Jorge-García D, Estruch-Guitart V, Aragonés-Beltrán P (2023) How geographical factors and decision-makers’ perceptions influence the prioritisation of ecosystem services: analysis in the Spanish rice field areas in RAMSAR Mediterranean wetlands. Sci Total Environ 869:161823. 10.1016/j.scitotenv.2023.161823 36708824 10.1016/j.scitotenv.2023.161823

[27] Leng M, Jin ZJ, Xiao XY et al (2022) Comparison of bacterial community structure in soil aggregates between natural karst wetland and paddy field. Huanjing Kexue 43:4353–4363. 10.13227/j.hjkx.202108277 35971731 10.13227/j.hjkx.202108277

[28] Li HY, Wang H, Tao XH et al (2021) Continental-scale paddy soil bacterial community structure, function, and biotic interaction. mSystems 6:e01128-21. 10.1128/msystems.01368-20 10.1128/mSystems.01368-20 PMC8547477 34546068

[29] Lin H, Peddada SD (2020) Analysis of compositions of microbiomes with bias correction. Nat Commun 11:3514. 10.1038/s41467-020-17041-7 32665548 10.1038/s41467-020-17041-7 PMC7360769

[30] Liu C, Ding N, Fu Q et al (2016) The influence of soil properties on the size and structure of bacterial and fungal communities along a paddy soil chronosequence. Eur J Soil Biol 76:9–18. 10.1016/j.ejsobi.2016.06.002

[31] Liechty Z, Santos-Medellín C, Edwards J et al (2020) Comparative analysis of root microbiomes of rice cultivars with high and low methane emissions reveals differences in abundance of methanogenic archaea and putative upstream fermenters. mSystems 5:e01128-20. 10.1128/msystems.00897-19 10.1128/mSystems.00897-19 PMC7029222 32071162

[32] Liu C, Cui Y, Li X, Yao M (2021) Microeco: an R package for data mining in microbial community ecology. FEMS Microbiol Ecol 97:fiaa255. 10.1093/femsec/fiaa255 33332530 10.1093/femsec/fiaa255

[33] Ma Y, Wang J, Liu Y et al (2023) *Nocardioides*: “Specialists” for hard-to-degrade pollutants in the environment. Molecules 28:7433. 10.3390/molecules28217433 37959852 10.3390/molecules28217433 PMC10649934

[34] Oksanen J, Simpson G, Blanchet F et al (2024) *vegan: Community Ecology Package*. R package version 2.6–8. https://vegandevs.github.io/vegan/

[35] Olsen SR, Sommers LE (1982) Phosphorus. In: AL (ed) methods of soil analysis part 2 chemical and microbiological properties, American Society of Agronomy, Soil Science Society of America, Madison, 403–430. 10.2134/agronmonogr9.2.2ed

[36] Parks DH, Chuvochina M, Rinke C, Mussig AJ, Chaumeil PA, Hugenholtz P (2022) GTDB: an ongoing census of bacterial and archaeal diversity through a phylogenetically consistent, rank normalized and complete genome-based taxonomy. Nucleic Acids Res 50:D785–D794. 10.1093/nar/gkab776 34520557 10.1093/nar/gkab776 PMC8728215

[37] Pielou EC (1966) The measurement of diversity in different types of biological collections. J Theor Biol 13:131–144. 10.1016/0022-5193(66)90013-0

[38] Qian H, Zhu X, Huang S et al (2023) Greenhouse gas emissions and mitigation in rice agriculture. Nat Rev Earth Environ 4:716–732. 10.1038/s43017-023-00482-1

[39] Quast C, Pruesse E, Yilmaz P et al (2013) The SILVA ribosomal RNA gene database project: Improved data processing and web-based tools. Nucleic Acids Res 41:D590–D596. 10.1093/nar/gks1219 23193283 10.1093/nar/gks1219 PMC3531112

[40] Ribas MP, García-Ulloa M, Espunyes J, Cabezón O (2023) Improving the assessment of ecosystem and wildlife health: Microbiome as an early indicator. Curr Opin Biotechnol 81:102923. 10.1016/j.copbio.2023.102923 36996728 10.1016/j.copbio.2023.102923

[41] Scarlett K, Denman S, Clark DR et al (2021) Relationships between nitrogen cycling microbial community abundance and composition reveal the indirect effect of soil pH on oak decline. ISME J 15:623–635. 10.1038/s41396-020-00801-0 33067585 10.1038/s41396-020-00801-0 PMC8027100

[42] Shannon CE, Weaver W (1949) The mathematical theory of communication. University of Illinois Press, Champaign. 10.1002/j.1538-7305.1948.tb01338.x

[43] Simpson EH (1949) Measurement of diversity. Nature 163:688. 10.1038/163688a0

[44] Tao K, Kelly S, Radutoiu S (2019) Microbial associations enabling nitrogen acquisition in plants. Curr Opin Microbiol 49:83–89. 10.1016/j.mib.2019.10.005 31733615 10.1016/j.mib.2019.10.005

[45] Thompson LR, Sanders JG, McDonald D et al (2017) A communal catalogue reveals Earth’s multiscale microbial diversity. Nature 551:457–463. 10.1038/nature24621 29088705 10.1038/nature24621 PMC6192678

[46] Timmis K, Ramos JL (2021) The soil crisis: the need to treat as a global health problem and the pivotal role of microbes in prophylaxis and therapy. Microb Biotechnol 14:769–797. 10.1111/1751-7915.13771 33751840 10.1111/1751-7915.13771 PMC8085983

[47] UNE 77325:2003. *Soil quality – Determination of total nitrogen content by dry combustion (elemental analysis)*. European Standards. https://www.en-standard.eu/une-77325-2003-soil-quality-determination-of-total-nitrogen-content-by-dry-combustion-elemental-analysis/?srsltid=AfmBOooHSI7fFciGkY_gDmWmXug8kYBYr4ISG2pX_pNCoLqUJs-13uBC

[48] Wiegand S, Jogler M, Jogler C (2018) On the maverick Planctomycetes. FEMS Microbiol Rev 42:739–760. 10.1093/femsre/fuy029 30052954 10.1093/femsre/fuy029

[49] Wilhelm RC, Amsili JP, Kurtz KS et al (2023) Ecological insights into soil health according to the genomic traits and environment-wide associations of bacteria in agricultural soils. ISME Commun 3:1. 10.1038/s43705-022-00209-1 37081121 10.1038/s43705-022-00209-1 PMC9829723

[50] Wu X, Peng J, Liu P et al (2021) Metagenomic insights into nitrogen and phosphorus cycling at the soil aggregate scale driven by organic material amendments. Sci Total Environ 785:147329. 10.1016/j.scitotenv.2021.147329 33940418 10.1016/j.scitotenv.2021.147329

[51] Yatsunenko T, Rey F, Manary M et al (2012) Human gut microbiome viewed across age and geography. Nature 486:222–227. 10.1038/nature11053 22699611 10.1038/nature11053 PMC3376388

[52] Zhang Y, Li Q, Chen Y et al (2019) Dynamic change in enzyme activity and bacterial community with long-term rice cultivation in mudflats. Curr Microbiol 76:361–369. 10.1007/s00284-019-01636-5 30684025 10.1007/s00284-019-01636-5

