# Supplementary material for: Soil Bacteriome Shifts along a Cultivation Gradient in Southwestern Spanish Wetlands
Source: Microb Ecol. 2025 Nov 29;89:16. doi: 10.1007/s00248-025-02660-8 (PMC12789212; doi:10.1007/s00248-025-02660-8)
Supplement: Supplementary file 1 [file 248_2025_2660_MOESM1_ESM.docx]

**Supplementary Material**

**Supplementary Table S1**. GPS coordinates of the different plots used for the prokaryotic bacteriome analysis.

| **Plot** | **Latitude** | **Longitude** |
| --- | --- | --- |
| M-1 | 37º5'47.4"N | 6º9'20.232"W |
| M-2 | 37º5'57.25"N | 6º10'7.432"W |
| M-3 | 37º6'53.266"N | 6º10'2.543"W |
| M-4 | 37º6'34.38"N | 6º10'47.68"W |
| M-5 | 37º7'24.755"N | 6º10'23.686"W |
| CA-1 | 37º3'33.012"N | 6º13'6.676"W |
| CA-2 | 37º2'0.204"N | 6º13'34.59"W |
| CA-3 | 37º1'21.67"N | 6º16'3.083"W |
| CA-4 | 37º2'39.797"N | 6º14'13.513"W |
| CA-5 | 37º3'27.684"N | 6º15'24.563"W |
| PN-1 | 37º6'34.999"N | 6º24'52.441"W |
| PN-2 | 37º6'35.33"N | 6º24'54.71"W |
| PN-3 | 37º6'35.48"N | 6º24'53.61"W |
| PN-4 | 37º6'35.36"N | 6º24'51.64"W |
| PN-5 | 37º6'37.29"N | 6º24'51.40"W |
| M: Mínima 2 (80 years of cultivation history); CA: Cantarita (25 years of cultivation history); PN (Doñana National Park) | | |

**Supplementary Table S2. Physicochemical parameters of cultivated and natural soils in the Guadalquivir Marshes.**

| **Sample*** | **pH**  **(Ext 1:2:5)** | **Conductivity**  **Ext 1:5)**  **(mS/cm)** | **Organic C**  **(%)** | **Organic**  **Matter**  **(%)** | **CaCO_3_**  **(%)** | **Total C**  **(%)** | **Total N**  **(%)** | **Ammonium**  **(mg/kg)** | **Nitrate**  **(mg/kg)** | **Available**  **Phosphorus**  **(mg/kg)** | **Potassium (mg/kg)** |  |
| --- | --- | --- | --- | --- | --- | --- | --- | --- | --- | --- | --- | --- |
| M-1 | 8.15 | 0.403 | 2.01 | 3.47 | 23.6 | 4.84 | 0.208 | 4.90 | 6.60 | 24.5 | 875 |  |
| M-2 | 7.79 | 1.010 | 1.60 | 2.76 | 24.6 | 4.56 | 0.190 | 5.00 | 4.20 | 8.90 | 999 |  |
| M-3 | 8.13 | 0.556 | 1.28 | 2.21 | 21.7 | 3.88 | 0.186 | 4.70 | 13.9 | 18.7 | 907 |  |
| M-4 | 7.93 | 0.628 | 2.55 | 4.40 | 24.1 | 5.43 | 0.250 | 2.90 | 14.3 | 16.7 | 886 |  |
| M-5 | 8.14 | 0.410 | 1.62 | 2.79 | 18.4 | 3.83 | 0.194 | 1.80 | 10.9 | 15.1 | 873 |  |
| CA-1 | 7.86 | 0.910 | 1.84 | 3.17 | 17.9 | 3.98 | 0.208 | < 0,20 | 19.4 | 19.9 | 953 |  |
| CA-2 | 8.01 | 0.571 | 1.32 | 2.28 | 21.1 | 3.85 | 0.163 | 3.70 | 6.00 | 9.90 | 950 |  |
| CA-3 | 8.03 | 0.741 | 1.38 | 2.38 | 17.7 | 3.50 | 0.171 | < 0,20 | 14.9 | 9.60 | 882 |  |
| CA-4 | 8.07 | 0.594 | 1.77 | 3.05 | 12.4 | 3.25 | 0.203 | 1.10 | 14.5 | 30.4 | 856 |  |
| CA-5 | 8.19 | 0.665 | 1.75 | 3.02 | 12.0 | 3.19 | 0.209 | 4.40 | 16.1 | 25.5 | 1017 |  |
| PN-1 | 8.66 | 0.160 | 0.903 | 1.56 | 14.1 | 2.59 | 0.113 | 1.10 | 1.00 | 8.30 | 802 |  |
| PN-2 | 8.55 | 0.175 | 1.37 | 2.36 | 8.38 | 2.37 | 0.162 | 1.30 | 0.800 | 13.5 | 999 |  |
| PN-3 | 8.70 | 0.423 | 0.788 | 1.36 | 8.39 | 1.80 | 0.117 | 1.10 | 1.60 | 6.30 | 1097 |  |
| PN-4 | 8.69 | 0.167 | 2.09 | 3.60 | 9.10 | 3.18 | 0.246 | 5.00 | 3.90 | 9.30 | 1322 |  |
| PN-5 | 8.26 | 0.228 | 2.33 | 4.02 | 7.78 | 3.27 | 0.267 | 3.40 | 2.30 | 18.5 | 1097 |  |
| M: Mínima 2 (80 years of cultivation history); CA: Cantarita (25 years of cultivation history); PN (Doñana National Park) | | | | | | | | | | | | |

Supplementary Figure S1. Location of the sampling points from the three studied areas.

**Supplementary Figure S2.** Relative abundance of dominant bacterial families in natural (Doñana) and cultivated soils (Cantaria and Mínima 2). Statistically significant differences, identified using Analysis of Compositions of Microbiomes with Bias Correction (ANCOM-BC2), are indicated by different letters (“a” and “b”).

**Supplementary Figure S3.** Random forest analysis identifying bacterial taxa that discriminate among the three study areas. Taxa with the highest discriminatory power include SBR1031 and *Anaerolineaceae* (cultivated soils) and *Euzebyaceae* and *Geminicoccaceae* (natural soils). A) MeanDecreaseGini score, indicating the most significant taxa in each condition; B) Relative abundance of the taxa identified in A).

Supplementary Figure S4. Alpha diversity analysis represented by Shannon and Simpson biodiversity indices, and Pielou’s evenness. Samples significatively different after an analysis of variance (ANOVA) are indicated with “a” and “b” letters.

Supplementary Figure S5. Pairwise comparison soils using Wilcoxon rank sum test and FDR correction with BH method for Observed ASVs, Shannon, Simpson biodiversity indices and Pielou’s evenness. Significant differences are represented by asterisks.

Supplementary Figure S6. Correlation analysis of physicochemical parameters in the different study sites.

Supplementary Figure S7. Permutation test assessing correlation significance among genus-level communities and physicochemical parameters. Significance: Spearman ρ = 0.01 (**), ρ = 0.05 (*).
